# Case of Fracture of the Hip-Joint, with Other Severe Injuries, Treated without Splints

**Published:** 1867-02

**Authors:** Theodore W. Fisher

**Affiliations:** Boston


*Case of Fracture of the Hip-Joint, with other Severe Injuries, Treated without Splints.* By THEODORE W. FISHER, M.D., Boston.

J. T., aged 13 years, a healthy boy, of Irish parentage, April 14th, 1862, fell from the fourth story upon a plank walk.

He was taken up insensible, but had revived when I saw him, ten minutes after the accident.

I found a compound, comminuted fracture into the right elbow-joint, with dislocation of the ulna and radius outwards. The external wound was two inches in length, and through it could be seen the whole articulation. Both radius and ulna were broken and much displaced, two inches above the wrist. The head of the radius was broken near the left wrist. One or more ribs were broken, on the right side, wounding the pleura and producing emphysema of the parietes of that side. There was distinct crepitus over the right trochanter, with eversion of the foot, and shortening to the extent of an inch. The shock was not very severe, and the pulse 88, and of fair strength. He suffered most from efforts to cough, which gave much pain, and a binder was applied to support the chest.

He was seen three hours after the accident by Dr. H. G. Clark, in consultation. The patient was etherized, fragments of bone were removed from the elbow, and dislocations reduced. An internal angular splint was loosely applied to the right arm, which was supported by a pillow, and water dressing applied. Straight splints were used for the wrists. Extension was applied to right foot by a brick attached in the usual manner to the leg, and counter-extension by strips of sheets, on each side of the perinæum, fastened to the head of the bed. Beef-tea and brandy were ordered in moderate doses. The reaction came on in the evening. Pulse 110. Brandy discontinued.

*April 15th.* Has slept none, and pulse 120. Hip very painful, with constant tendency to eversion, which was counteracted by a broad strip of plaster, around the thigh, attached to a weight hanging over the left side of the bed. No extension of emphysema. Slight bloody expectoration. Morphine gr. $\frac{1}{8}$ at bed time.

*16th.* Slept six hours. Pulse 116, full and quick. Breathes more easily.

*17th.* Slept well. Eats well. Pulse 128.

*18th.* Pulse 112. Respirations 28. Elbow swollen.

*19th.* Swelling and discoloration in right groin noticed.

*20th.* Pale and chilly. Emphysema disappearing. Bears extension well. Pulse 100. Wound in elbow discharges freely.

*29th.* Complains of severe stinging pain in second, third, and fourth fingers of right hand. Pulse 100. Ordered quinine, gr. j., three times a-day.

*May 10th.* Etherized, and two openings made in the elbow, discharging a small quantity of pus. Poultice ordered.

*29th.* Etherized, and a free opening made near inner condyle. Considerable pus and a small fragment discharged.

*June 1st.* Weak and irritable. Pulse 120. Most of weight removed from leg. Nourishing diet urged on him.

*15th.* Doing well. Encourage motion of leg; to be kept flexed part of time by means of a sling.

*26th.* Sits up and walks a little.

*July 14th.* Three months after the accident. Walks with a slight limp only. Perfect motion in left wrist. Good union of radius and ulna of right arm, with increasing motion of the hand. Elbow stiff, and fixed at a convenient angle.

The patient eventually walked without lameness, and recovered a slight motion in his elbow.

